# Structure of Amphiphilic Terpolymer Raspberry Vesicles †

**DOI:** 10.3390/polym9070275

**Published:** 2017-07-09

**Authors:** Yingying Guo, Luca di Mare, Robert K. Y. Li, Janet S. S. Wong

**Affiliations:** 1 Department of Mechanical Engineering, Imperial College London, London SW 7 2AZ, UK; yingying.guo@imperial.ac.uk; 2 Department of Engineering Science, University of Oxford, Southwell Thermofluids Laboratory, Oxford OX2 OES, UK; 3 Department of Materials Science and Engineering, City University of Hong Kong, Hong Kong, China; aprkyl@cityu.edu.hk

**Keywords:** raspberry vesicles, DPD simulations, star terpolymer, equilibrium structures, cargo release

## Abstract

Terpolymer raspberry vesicles contain domains of different chemical affinities. They are potential candidates as multi-compartment cargo carriers. Their efficacy depends on their stability and load capacity. Using a model star terpolymer system in an aqueous solution, a dissipative particle dynamic (DPD) simulation is employed to investigate how equilibrium aggregate structures are affected by polymer concentration and pairwise interaction energy in a solution. It is shown that a critical mass of polymer is necessary for vesicle formation. The free energy of the equilibrium aggregates are calculated and the results show that the transition from micelles to vesicles is governed by the interactions between the longest solvophobic block and the solvent. In addition, the ability of vesicles to encapsulate solvent is assessed. It is found that reducing the interaction energy favours solvent encapsulation, although solvent molecules can permeate through the vesicle’s shell when repulsive interactions among monomers are low. Thus, one can optimize the loading capacity and the release rate of the vesicles by turning pairwise interaction energies of the polymer and the solvent. The ability to predict and control these aspects of the vesicles is an essential step towards designing vesicles for specific purposes.

## 1. Introduction

Vesicles are nanoaggregates capable of encapsulating chemicals, including solvents, in a closed structure. They are important because they can be used as nanoreactors or carriers [1,2,3,4]. While vesicles are commonly formed using lipids and surfactants, interest in vesicles formed using block copolymer has increased recently since they offer a wide range of morphologies and chemical affinities [5,6]. Among block copolymers, ABC star terpolymers are particularly attractive because, compared to linear block copolymers, they form more stable aggregates [1,2,3] and their aqueous solutions have lower viscosity [4,5,7]. ABC star terpolymers are tri-block copolymers that consists of A-, B- and C-blocks of different chemistry. To date, star block terpolymers including [poly(ethylethylene)][poly(ethylene oxide)][poly(perfluoropropylene oxide)] PEO-PEE-PFPO [8], [poly(styrene)][poly(l-lactide)][poly(tert-butyl methacrylate)] PS-MI(PLLA)-P(S/tBMA) [9], [poly(3-ethyl-3-oxetanemethanol)]-star-[poly(ethylene glycol)] HBPO-star-PEO [10], and [poly(methyl methacrylate)][poly(2-(diethylamino) ethyl methacrylate)][poly(poly(ethylene glycol)methyl ether methacrylate)] PMMA-PDEA-PPEGMA [11] have successfully been used to form vesicles.

The variety and properties of aggregates formed by ABC terpolymers are related to their structures. Star ABC terpolymers are block copolymers composed of blocks connected at one common point. The blocks are mutually immiscible and have different solvophobicities. The strong repulsion among the blocks causes ABC terpolymers to self-assemble into aggregates with segregated A, B, and C nanodomains. These aggregates have a variety of structures, many of which cannot be observed in solutions of linear copolymers. Polygonal bilayers [8], nanostructured vesicles [8,12], segmented network structures [13], bead-on-string worm micelles [14], and segmented semivesicles [12] have all been reported or predicted.

The equilibrium structures formed by star ABC terpolymers in selective solvents depend on concentration [13,15,16], interaction energies among blocks and the solvent (Flory-Huggins interaction parameter χ) [9,12,14,15,17], pH [18], and temperature [11,19]. Phase diagrams of miktoarm star copolymer melts [20] and solutions [21] have been produced numerically to explore the influence of the interaction parameter and solvent quality [9,12,14,15,17] on the self-assembly behaviour of miktoarm star copolymers in solution.

The length of individual blocks of block copolymer determines the morphology and stability of the equilibrium aggregates [8,12,13,14,15,17,22,23]. Terpolymers with a longer solvophilic block form aggregates with discrete multi-domain cores. Decreasing the solvent quality leads to the formation of cylindrical or disk-like micelles [17]. As the length of the solvophilic block decreases, the aggregates change from raspberry/hamburger micelles, to segmented wormlike micelles and, finally, nanostructured bi-layers. Studies to this effect have been conducted both experimentally, on PEO-PEE-PFPO [8,23], and numerically [12,13,14,15,17,22]. When the bilayers are sufficiently large, they can fold and form vesicles [22]. These vesicles are referred to as raspberry vesicles, as their surfaces consist of segregated solvophobic nanodomains in a solvophilic matrix and resemble the surface of a raspberry. The equilibrium morphology of vesicles formed by ABC terpolymers is also affected by the length ratio between the solvophobic blocks [12,15,23]. Chemicals with various affinities to each domain could be transported concurrently by raspberry vesicles.

It has been shown that miktoarm star ABC terpolymer with one longer solvophobic arms but shorter solvophilic arms can self-assemble into spherical vesicles in a wide range of solvent conditions [17]. The system presented in this work corresponds to amphiphilic μ-[poly(ethylethylene)][poly(ethylene oxide)][poly(perfluoropropylene oxide)] (μ-EOF) star terpolymer in aqueous solutions. The three blocks poly(ethylethylene) (PEE), poly(ethylene oxide) (PEO), and poly(perfluoropropylene oxide) (PFPO), and the water molecules are represented by four kinds of beads :A, B, C, and S, respectively. The solvophilic block is, therefore, block B, while block C is the most solvophobic. μ-EOF star terpolymers are known to form nanostructured vesicles [8,22,23]. Simulations in this work use A_12_B_6_C_2_ as the model polymer. The chosen length ratio corresponds to a solvophilic weight fraction of 35% (±10%) and is known to promote vesicle formation [3]. Furthermore, a long solvophobic A-block promotes the formation of aggregates with a raspberry-like shell [23,24]. As a result, vesicles are the equilibrium aggregates in many test conditions in this work.

Using our model system, the equilibrium structure of the aggregates is explored using dissipative particle dynamics (DPD) simulations. Effects of the interactions among beads and solvents and that of polymer concentration are investigated. Surface morphologies and structures of aggregates are examined. The thermodynamics that governs the equilibrium aggregate structures is discussed.

As vesicles formed with star terpolymers can be efficient carriers, it is necessary to identify conditions under which they are the equilibrium structures and to study how their encapsulation efficiency and properties vary with their morphological details and with the environment. A state diagram showing the equilibrium aggregate structure is constructed to ascertain the optimal conditions for vesicle formation. The ability of the aggregates to encapsulate solvent is explored. This paper sheds light on how polymer concentration and interaction energies among various blocks of a terpolymer and with the solvent govern the equilibrium aggregate structures and their properties. The ability to predict and control these aspects of the vesicles is an essential step towards designing vesicles for specific purposes [3].

The remainder of the paper will present the methodology applied and the major findings of this study. Note, figures, and tables denoted by the letter “S” can be found in the accompanying Supplementary Material available online.

## 2. Materials and Methods 

Dissipative particle dynamics (DPD) simulations are used to study the spontaneous self-assembly of ABC star terpolymers in a solvent S. DPD is a particle-based mesoscopic simulation technique first introduced by Hoogerbrugge and Koelman [25]. The terpolymer is coarse-grained and each DPD particle (or bead) is a collection of molecules. The position $x_{i}$ and velocity $v_{i}$ of each DPD particle is governed by Newton’s equation of motion:

(1) $$\frac{d{\vec{x}}_{i}}{dt}\;=\;{\vec{v}}_{i},\;m\frac{d{\vec{v}}_{i}}{dt}\;=\;{\vec{f}}_{i}$$

where $m_{i}$ is the particle mass, ${\vec{f}}_{i}$ is the total force exerted on each particle i and consists of four components: conservative force ${\vec{F}}_{ij}^{C}$, dissipative force ${\vec{F}}_{ij}^{D}$, random force ${\vec{F}}_{ij}^{R}$, and spring force ${\vec{F}}_{ij}^{S}$. The first three forces are pairwise contributions and become effective when the distance between two beads i and j is within the cut-off radius $r_{c}\;=\;1$. The spring force ${\vec{F}}_{ij}^{S}$ is a harmonic spring force acting on consecutive (bonded) beads. The conservative force ${\vec{F}}_{ij}^{C}$ between nonbonded beads is a soft-repulsive force and is given by:

(2) $${\vec{F}}_{ij}^{C}\;=\;\{\begin{array}{c} a_{ij}\left(1\;-\;\frac{r_{ij}}{r_{c}}\right){\hat{r}}_{ij}\;\mathrm{if}\;r_{ij}\;<\;r_{c}\\ 0\;\mathrm{otherwise} \end{array}$$

where ${\vec{r}}_{ij}\;=\;{\vec{r}}_{i}\;-\;{\vec{r}}_{j}$, $r_{ij}\;=\;\left|{\vec{r}}_{ij}\right|$, ${\hat{r}}_{ij}\;=\;{\vec{r}}_{ij}/\left|{\vec{r}}_{ij}\right|$ and $a_{ij}$ is the repulsion between beads i and j. The DPD soft-repulsive force allows simulations of larger length- and time-scales as compared to the 6–12 Lennard Jones potential used in non-equilibrium molecular dynamics (NEMD) computations. The combination of dissipative force ${\vec{F}}_{ij}^{D}$ and random force ${\vec{F}}_{ij}^{R}$ acts as a thermostat in the simulation. The two forces are evaluated as:

(3) $${\vec{F}}_{ij}^{D}\;=\;-{\gamma \omega }^{D}\left(r_{ij}\right)\left(\;{\vec{v}}_{ij}\cdot \;{\vec{r}}_{ij}\right){\hat{r}}_{ij}$$

(4) $${\vec{F}}_{ij}^{R}\;=\;-{\sigma \omega }^{R}\left(r_{ij}\right)\frac{\xi_{ij}}{\sqrt{\Delta t}}\;{\hat{r}}_{ij}$$

where Δt is the simulation time step, ${\vec{v}}_{ij}={\vec{v}}_{i}-{\vec{v}}_{j}$, σ, and γ respectively represent the amplitude of ${\vec{F}}_{ij}^{D}$ and ${\vec{F}}_{ij}^{R}$, $\omega^{D}\left(r_{ij}\right)$ and $\omega^{R}\left(r_{ij}\right)$ are the weight functions, and $\xi_{ij}$ is a random number with zero mean and unit variance. In order to satisfy the equilibrium Gibbs-Boltzmann distribution and the fluctuation-dissipative theorem, the following two relationships are required:

(5) $$\omega^{D}\left(r_{ij}\right)\;={\left(\omega^{R}\left(r_{ij}\right)\right)}^{2}$$

(6) $$\sigma^{2}\;=\;2\gamma k_{b}T$$

In our simulations, σ is chosen to be 3 [26] and temperature $k_{b}T$ is chosen to be 1. According to Groot and Warren [27], the weight function is expressed as:

(7) $$\omega^{D}\left(r_{ij}\right)=\{\begin{array}{c} {\left(1-\frac{r_{ij}}{r_{c}}\right)}^{2}\;\mathrm{if}\;r_{ij}<r_{c}\\ 0\;\mathrm{otherwise} \end{array}$$

This restricts the interaction among beads only with their neighbours. The dissipative force ${\vec{F}}_{ij}^{D}$ reduces the relative momentum between particles i and j. The random forces ${\vec{F}}_{ij}^{R}$ impart energy to the system. The spring force ${\vec{F}}_{ij}^{S}$ between bonded beads is given by:

(8) $${\vec{F}}_{ij}^{S}\;=\;\underset{j}{\sum }C\left(r_{ij}\;-\;r_{eq}\right){\hat{r}}_{ij}$$

where C is the spring constant and set as 100.0 [28], and the equilibrium bond length is $r_{eq}\;=\;0.86$. This value results in a slightly smaller distance between bonded particles than non-bonded ones, and the sum runs over all particles to which particle i is connected.

The integration of the equation of motion (Equation (1)) is carried out using the Velocity-Verlet algorithm [29] with a time step Δt = 0.04 in DPD units. The unit of time $\tau \;=\;r_{c}\sqrt{m/k_{b}T}$ corresponds to 10.45 ps in real time. Each time step then is 0.418 ps.

The interactions among blocks are varied with a coupling parameter λ:

(9) $$a_{ij}=\;\lambda a_{ij}^{0}\;+\;\left(1\;-\;\lambda \right)a_{ii}^{0}$$

The coupling parameter λ is introduced to allow the calculation of relative free energy associated with individual aggregates. This also provides an opportunity to investigate how changes in pair-wise interaction energies, in the form of varying λ, affect equilibrium aggregate structures and their properties. Details of the free energy calculation are given in Section 3.1. The maximum pairwise interactions $a_{ij}^{0}$ are recovered for λ=1. This corresponds to the interactions among PEE (A bead), PEO (B bead), PFPO (C bead), and water (S bead). The values $a_{ij}^{0}$ are reported in Table 1.

The volumes of the repeat unit of PEE, PEO, and PFPO are 122.5, 65.2, and 149 ${Å}^{3}$ respectively [22]. All beads have similar size of 900 ${Å}^{3}$. This means there are 30 water molecules in one S bead, 7.3 PEE mer units in one A bead, 14 PEO mer units in one B bead, and six PFPO mer units in one C bead. Details on the coarse-grained method and the choice of $a_{ij}^{0}$ values can be found in [22].

The present DPD simulations focus on the effect of polymer concentration on the spontaneous vesicle formation of A_12_B_6_C_2_ star terpolymer in solutions made of S beads. Each polymer chain has 20 beads which consists of 12 A beads, six B beads, and two C beads. Unless explicitly stated, results presented are from simulations consist of 192,000 DPD beads in a simulation box of 40$r_{c}\times$ 40$r_{c}\times$ 40$r_{c}$ with periodic boundary conditions. This corresponds to a box length of 56 nm. The volume fraction of polymer in the solution $\varphi_{p}$ is defined as the total number of polymer beads divided by the total number of beads in the simulations. The simulation begins with a homogenous state. A minimum of 3 million DPD steps are taken to ensure equilibrium states are achieved. NVT ensemble is adopted in the simulation. Temperature is set at 298 K. $\varphi_{p}$ between 0.02 and 0.10 are investigated. This translates to 192–960 chains in the box and the corresponding pressures are 204 (20.7)–212 (21.5) atm (MPa), respectively. Simulations have also been conducted with a box size of 80$r_{c}\times$ 80$r_{c}\times$ 80$r_{c}$.

## 3. Analytical Methodologies

### 3.1. Thermodynamic Integration for Free Energies

Free energies are evaluated to identify the factors driving the aggregation of the terpolymers into micelles and vesicles. The free energy $G_{ij}\left(\eta \right)$ results in this paper are free energies of the solution of concentration $\varphi_{p}$ with respect to an ideal solution of terpolymers of the same concentration. The thermodynamic integration is performed by evaluating the integral:

(10) $$G_{ij}\left(\eta \right)\;=\;\int_{0}^{\eta }\frac{\partial U_{ij}}{\partial \lambda }d\lambda$$

The integrand $\frac{\partial U_{ij}}{\partial \lambda }$ represents the partial derivative of the interaction energy between species i and j with respect to the coupling parameter λ. $\frac{\partial U_{ij}}{\partial \lambda }$ is a function of the coupling parameter itself and can be evaluated by ensemble averaging at fixed values of λ. It is, therefore, possible to build an approximation of $\frac{\partial U_{ij}}{\partial \lambda }$ using Tchebychev polynomials and then perform the integration analytically on the polynomial approximation. Eighth-order polynomials are used to approximate the variation of $\frac{\partial U_{ij}}{\partial \lambda }$ with the varying λ. This procedure has the advantage of returning the variation of the free energy as a function of the interaction strength. This is equivalent to studying a family of solutions of increasing interaction strength rather than a single chemical system. The transition from a micelle to a vesicle is a second-order transition, as can be seen from the variation of internal energy shown in Supplementary Materials Figure S1. Thermodynamic integration can, therefore, be carried out across this transition.

### 3.2. Skin Characterisations 

**Scheme 1:**
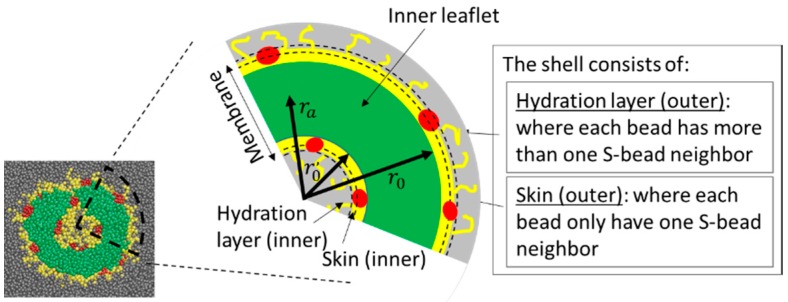
Definition of various terms used to describe the membranes. The colour codes for A-, B-, C-, and S-beads are green, yellow, red, and grey, respectively. $r_{0}$ is the minimum distance between the centre of mass of the aggregate and the hydrophilic B-beads located near the outer surface of the aggregate. $r_{0}^{\prime }$ is used, if the aggregate is a vesicle, to define the inner shell and is the maximum distance between the centre of mass of the aggregate and the B-beads near the inner surface. $r_{a}$ is the distance between the centre of mass to the average mid-plane of the membrane. The definition of the membrane excludes the hydrated layer.

The majority of the analysis carried out in this work concerns properties of surfaces of aggregates, which we refer to as “skin”. Take a vesicle as an example: a vesicle is made of a membrane, with a cavity inside. The membrane is made of three parts; the inner shell, which is adjacent to the cavity; the outer shell, which is the surface exposed to the surroundings; and the inner leaflet, which separates the two shells (see Scheme 1). The outer shell of a vesicle consists of A-, B-, and C-beads which (1) are in contact with S-beads and (2) are located more than $r_{0}$ units away from the center of mass of the aggregate. The definition of the inner shell is similar to that of the outer shell except, in this case, the beads on the inner shell must be at most $r_{0}^{\prime }$ away from the center of mass of the aggregate. $r_{0}$ ($r_{0}^{\prime }$) is the minimum (maximum) distance between the centre of mass of the aggregate and the hydrophilic blocks near its outer (inner) surface. The second condition is in place to avoid counting beads that are not on the surface, but have contact with S-beads that have permeated into the aggregate membrane. The shell is subdivided into two parts. The beads with only one S-neighbour form the skin of the aggregate. Those with more than one S-neighbour are part of the hydration layer. Beads in the membrane that have no contact with the S-bead form the inner leaflet of the membrane. A-, B-, C-, and S- beads in the membrane that do not belong to the shell, and S-beads in the centre cavity, form the core of the aggregate. For micelles, only the outer shell exists. The rest of the beads, including any trapped S-beads in a micelle, form its core.

The aggregate size $r_{a}$, excluding its hydration layer, is defined as:

(11) $$r_{a}\;=\;\sqrt{\frac{1}{3}\left(I_{1}\;+\;I_{2}\;+\;I_{3}\right)}$$

where $I_{1}<I_{2}<I_{3}$ are the principal moments of inertia of the aggregate with respect to its center of mass. The skin can be mapped in spherical coordinate (r,θ,ψ) where r is the distance from the center of mass, and θ and ψ are the longitude and latitude angles, referring to the principal directions of the tensor of inertia of the aggregate, as shown in Supplementary Materials Figure S2a.

## 4. Results and Discussion

### 4.1. General Descriptions of Equilibrium Aggregate Structures

**Figure 1:**
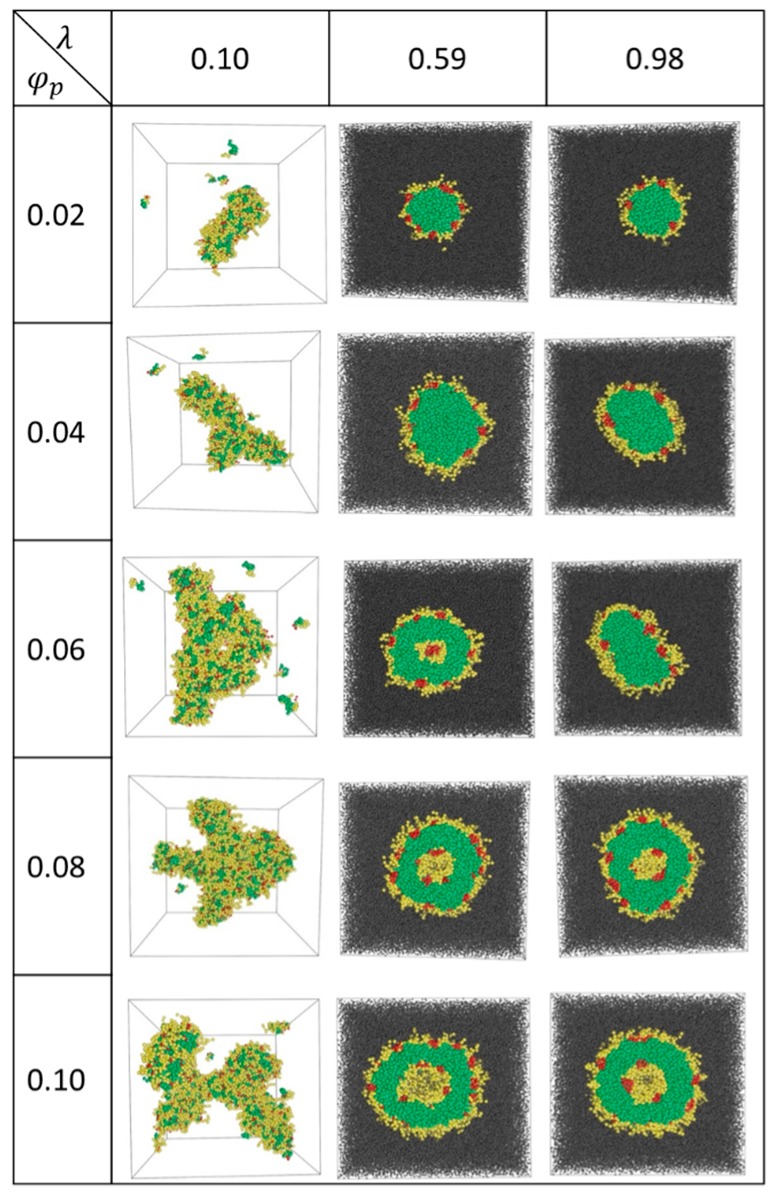
Aggregate structures for λ *=* 0.10, 0.59 and 0.98 at various $\varphi_{p}$. Green, yellow, red, and grey beads refer to A-, B-, C-, and S-beads, respectively. For λ *=* 0.10, S-beads (grey) are omitted for clarity.

The equilibrium aggregates formed at various values of λ and $\varphi_{p}$ are shown in Figure 1. At the lowest value of λ = 0.10, where the interactions among various beads are relatively weak, A_12_B_6_C_2_ star terpolymers form fragmented aggregates at low $\varphi_{p}$ and networks of rings and cylinders at high $\varphi_{p}$ (see Figure 1). In these cases, B- and C-beads (yellow and red beads in Figure 1) reside on the surface of the aggregates with relatively low surface coverage. As a result, many A-beads (green) are exposed to the solvent (S-beads omitted for cases with λ = 0.10). No distinct C-domain is formed.

At λ = 0.98, spherical raspberry micelles with A-bead cores (green) are formed at low $\varphi_{p}$. These micelles have shells that are made mainly of B-beads (yellow) with small C-bead domains (red) that give these micelles the appearance of raspberries. Some B-bead segments on the shell are hydrated by S-beads. The hydrated B-beads extend out of the skin into the solvent space (grey) as yellow ‘hair’ growing out of the aggregates. The shapes of these raspberry micelles change from spherical ($\varphi_{p}$ = 0.02) to disc-like ($\varphi_{p}$ = 0.06) as $\varphi_{p}$ increases. The observation that micelles become less circular as they approach the micelle-vesicle boundary is general and is quantified in the Supplementary Material Figure S3 (see also Section 4.2). As $\varphi_{p}\;\geq$ 0.08, spherical nanostructured bilayer vesicles form. The vesicles contain encapsulated S-beads (grey) in the cavity. The membrane consists of an inner and an outer shells with skins made of B-bead layers (yellow) decorated with C-bead (red) domains. The skins are separated by the inner leaflet of the membrane made of A-beads (green).

Similar structures are observed for intermediate values of λ. As an example, for λ = 0.57, aggregates change from micelles to vesicles as $\varphi_{p}$ increases, with one exception: at $\varphi_{p}\;=$ 0.06, a semivesicle is observed. A semivesicle is formed due to segregation of segments in a micelle, giving rise to a solvophobic C-bead center core. At $\varphi_{p}\;\geq$ 0.08, vesicles are the equilibrium structures.

The equilibrium structures at high λ have been observed both in experiments [8] and simulations [22]. The transition from micelles to vesicles with increasing λ [12,30] and $\varphi_{p}$ is consistent with reports from the literature [31]. The network structure presents at the lowest values of λ are rarely seen experimentally. This is probably due to the lack of terpolymers with such weak repulsive interactions.

### 4.2. State Diagram

**Figure 2:**
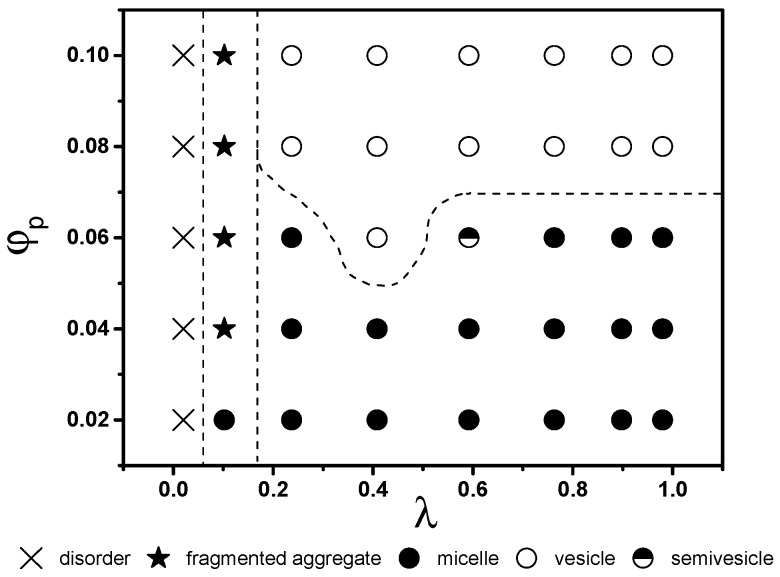
The effects of polymer concentration and interaction energies among beads on the equilibrium aggregate structures. Solid and open symbols correspond to micellar and vesicular aggregates, respectively.

To illustrate the effect of interactions among various beads in the star terpolymer and the solvent, equilibrium structures for various $\varphi_{p}$ and λ are presented in Figure 2 in the form of a state diagram. Note equilibrium structures of low λ solutions obtained using initializations with homogeneous systems are identical to those obtained from the final states of simulations at higher λ. This verifies that results shown in Figure 1 and Figure 2 are independent of transformation paths.

At very low λ, where interactions among all beads and the solvent are equal or relatively small, the solvent and the terpolymer mix well (disordered state, × in Figure 2). Most terpolymer chains remain as individual chains. An increase in λ favours self-assembly of terpolymers driven by repulsion among beads. At λ ~ 0.1, fragmented or branched aggregates are observed (★ in Figure 2). These aggregates are only stable at low λ. At higher λ, two main types of aggregate structures emerge, micelles and vesicles (solid and open circles, respectively, in Figure 2). They divide the remaining part of the state diagram into two regions, with low and high $\varphi_{p}$ favouring the formation of micelles and vesicles, respectively. The boundary between micelles and vesicles is located approximately at $\varphi_{p}=$ 0.07. The value of $\varphi_{p}$ above which vesicle formation is observed exhibits a weak dependence on λ: at λ = 0.4, the vesicles are formed above $\varphi_{p}\;=\;0.05$. Note that, in this study, a change in concentration corresponds to a change in the total mass of the aggregates. Results in Figure 2, thus, suggest the existence of a critical mass for vesicle formation. This critical mass is approximately 670 chains. The smallest vesicles generated in computations on a system containing a total number of particles eight times larger have approximately 700 chains, supporting an existence of a critical mass.

The geometry (size and shape) of aggregates can be characterized by their principal moments of inertia $I_{1}$, $I_{2}$, and $I_{3}$. Note the hydration layers are excluded from consideration. These values indicate how mass is distributed within an aggregate. Hence, their ratios reveal information about aggregate shapes. Contours of $I_{2}$ and *I*_3_ normalized by $I_{1}$ ($I_{2}^{\prime }\;=\;\sqrt{\frac{I_{2}}{I_{1}}}$ and $I_{3}^{\prime }\;=\;\sqrt{\frac{I_{3}}{I_{1}}}$) are presented in Supplementary Materials Figure S3. For a spherical aggregate $I_{1}\;=\;I_{2}\;=\;I_{3}$, hence, $I_{2}^{\prime }\;=\;I_{3}^{\prime }\;=\;1$. Most vesicles (above the grey dash line in Supplementary Materials Figure S3) are nearly spherical. For micelles, however, $I_{2}^{\prime }\;\neq \;I_{3}^{\prime }$ in most cases. Depending on the ratio between $I_{2}^{\prime }$ and $I_{3}^{\prime }$, the micelles range from spherical to disc-like, cylindrical, and elliptical. $I_{2}^{\prime }$ and $I_{3}^{\prime }$ deviate most from 1 for those micellar aggregates formed near the micelle-vesicle boundary. This shows that the transition from micelle to vesicle involves substantial geometric adjustment of aggregates.

### 4.3. Free Energy

**Figure 3:**
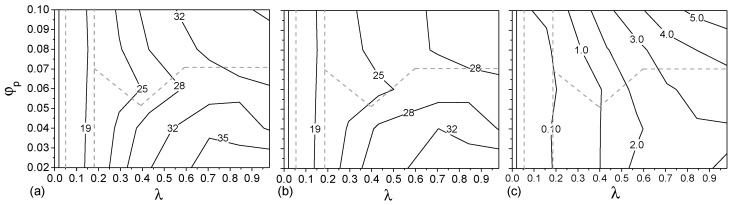
Relative (**a**) total free energy $F_{total}$; (**b**) total skin energy $F_{skin}$; and (**c**) core energy $F_{core}$ per terpolymer chain with respective to the ideal solution of aggregates formed at various $\varphi_{p}$. The dashed line shows the phase boundary.

The relative total free energy $F_{total}$ (excluding the hydration layer), total skin energy $F_{skin}$ (energy contributed by beads on the skins) and total core energy $F_{core}\;=\;F_{total}\;-\;F_{skin}$ per terpolymer chain with respect to the ideal solution were calculated as functions of $\varphi_{p}$ and λ and shown in the form of contour maps (see Figure 3). Note that the contributions for entrapped S-beads in the aggregates are included in these calculations.

The dominant contribution to the free energy for all clusters is $F_{skin}$. $F_{core}$ is never larger than 20% of the total free energy per chain. It is however an increasing fraction of the total free energy for larger vesicles (larger $\varphi_{p}$) and at higher values of λ. The micelle/vesicle stability boundary (grey dashed line, Figure 3) follows closely the contours of $F_{skin}.$ Hence, the relative stability of the micelle and vesicle phases is largely determined by their surface energy. Note the two phases have the same energy at the transition boundary, but the free energy of the micelle phase at concentrations higher than the critical concentration is larger than that of the corresponding vesicle phase. The rearrangement from micelles to vesicles allows $F_{skin}$ to decrease, at the expense of a small increase in $F_{core}$.

Further insight into the contributions to the free energy can be gained from Supplementary Materials Figure S4, showing the contributions of B-S, A-S, and C-S interactions to the total free energy of aggregates. A-S interaction dominates, and it exhibits a discontinuity during the micelle/vesicle transition due to rearrangement of polymers on the surface.

### 4.4. Morphology of Clusters

Various combinations of $\varphi_{p}$ and λ can result in geometrically similar aggregates, with subtle differences in bead distribution. Detailed structures and morphologies of equilibrated aggregates observed in Figure 1 and Figure 2 are described in this section. In this discussion, aggregates are divided into two parts, the shell and the core, as described in Section 3.2.

Information about aggregate sizes (excluding hydrated layers), in the form of average radii $r_{a}$, is presented in Supplementary Materials Figure S5a. $r_{a}$ increases with $\varphi_{p}$ since there are more terpolymers per aggregate. At a given $\varphi_{p}$, it is largely insensitive to λ. At $\varphi_{p}\;=$ 0.1, $r_{a}$ (solid squares, Supplementary Materials Figure S5b) decreases slightly with λ. This is inconsistent with how the radii of gyration $R_{g}$ of the terpolymer chains, and those of individual A-bead and B-bead segments, are affected by λ and $\varphi_{p}$, as shown in Supplementary Materials Figure S6. The size of B-bead segments (Supplementary Materials Figures S6b) is relatively constant while that of A-bead segments (Supplementary Materials Figure S6c) increases slightly with λ. Since the aggregates are made mainly of A-beads, the increase in size of A-bead segments translates to an increase in $R_{g}$ of polymer chains (Supplementary Materials Figure S6a). However $r_{a}$ decreases with λ (see Supplementary Materials Figure S5a). Note, as λ decreases, the interactions among all beads become more favourable, so that S-beads swell the membrane more (see Section 4.5.2 for details). These vesicles formed at low λ encapsulate more S-beads as their vesicle membranes are also thinner (see difference between solid squares and open circles, Supplementary Materials Figure S5b), resulting in larger $r_{a}$. This suggests that the size of vesicles of a given mass is controlled by the strength of the interaction between the solvent and polymer beads.

**Figure 4:**
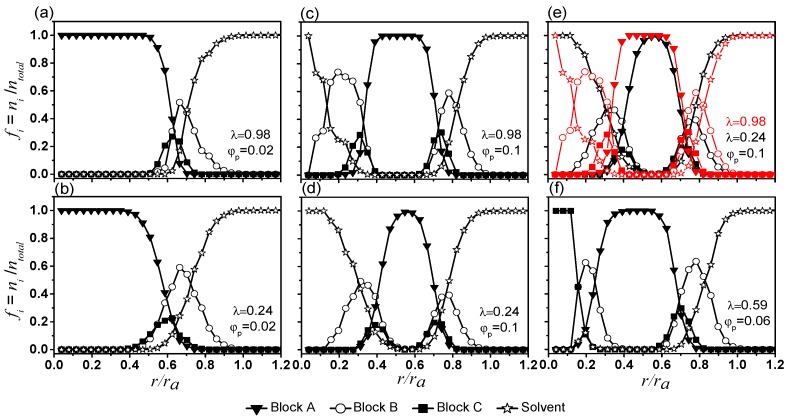
Composition profiles of aggregates (including skins and hydrated layers) formed under various conditions. Note the horizontal axis is the distance r from the center of mass of the aggregate normalized by the radius of the aggregate $r_{a}$. The vertical axis is the number fraction of bead i, $f_{i}$, in a particular $r/r_{a}$ and is the ratio of numbers of bead i to the total numbers of beads situated at a distance r away from the centre of mass.

Composition profiles, showing how fractions of various beads $f_{i}$ (i = A, B, C, and S-beads) change from the centre of mass of micelles ($r/r_{a}\;=$ 0) to their peripheries for $\varphi_{p}=$ 0.02 are presented in Figure 4a,b for λ = 0.98 and 0.24 respectively. All micelles feature an A-bead (inverted triangles) center surrounded by a shell made mainly of B- (circles) and C-beads (squares). The B-beads (circles) and C-beads (squares) are most abundant at $\frac{r}{r_{a}}\approx \;0.6-0.7$. Lower values of λ favour hydration of the B-beads (circles in Figure 4b). In addition, S-beads penetrate slightly deeper into the A-bead center (stars in Figure 4b). Overall, the effect of λ on the details of micelle morphology and size are small.

Vesicles contain S beads in their cavities. Their inner and outer shells have properties generally similar to shells of micelles (see Figure 4c,d for $\varphi_{p}\;=$ 0.1 λ = 0.98 and 0.24, respectively). The locations of B beads (circles) and S-beads (stars) overlap, suggesting a large portion of the B-beads are hydrated (see also Figure S7a,b in the Supplementary Information for compositional profiles of vesicles, excluding their shells). For the inner shells, $f_{B}$ (circles) and $f_{C}$ (squares) peak near $r/r_{a}\;\approx$ 0.2–0.3 and 0.3–0.4 respectively. Some hydrated B-beads extend to the centre of the aggregates. For outer shells, $f_{B}$ and $f_{C}$ peak near $r/r_{a}\;\approx$ 0.8 and 0.7, respectively. Similar to that of the inner shell, B-beads at the outer shell are highly hydrated and $f_{B}$ are higher than $f_{C}$.

An inner leaflet made of A-beads (inverted triangles, with $f_{A}\;\sim$ 1) separates the inner and outer shells of vesicles. A comparison of profiles from high (red) and low (black) λ is shown in Figure 4e. For $\varphi_{p}=$ 0.1 the thickness of the inner leaflet increases with λ (see also Supplementary Materials Figure S5b). The results in Figure 4a,b,e and Supplementary Materials Figure S5b suggest that an increase in the strength of interactions among various beads (by increasing λ) gives rise to vesicles with thicker and more well-defined bilayer membrane. They have a thicker solvated B-bead layer, a thicker A-bead inner leaflet, and an inner- and an outer- shells that are more effective in isolating the inner leaflet A-beads from S-beads.

Profiles of vesicles from $\varphi_{p}\;=$ 0.1 with λ ≥ 0.41 show similar composition profiles (see Supplementary Materials Figure S8a–d) and they are of similar sizes (see Supplementary Materials Figure S5a), suggest that these vesicles might have already reached their densest possible packing. Vesicles are also formed at lower $\varphi_{p}$ (see Supplementary Materials Figure S8f–j). While these vesicles are smaller (see Supplementary Materials Figure S5a), the effect of λ on the structure of these vesicles are similar to those formed with $\varphi_{p}\;=$0.1.

For $\varphi_{p}=$ 0.06 and λ = 0.59, a semivesicle is formed (see Figure 1 and Figure 4f). Semivesicles have been observed in computational simulations [32,33,34]. The difference between a vesicle and the semivesicle is clearly seen in Figure 4d,f. While the semivesicle has an inner and an outer shell, its centre is made of C-beads (squares, $f_{c}\;=$ 1) instead of S-beads as seen in vesicles.

### 4.5. Properties of Shells of Aggregates 

#### 4.5.1. Skin Morphology

The properties of the skins of the aggregates are discussed and results are presented in the form of maps in spherical coordinates. The definition of the spherical coordinates ψ-θ and the selection criteria for the shell and skin beads are shown diagrammatically in Supplementary Materials Figure S2a and in Scheme 1 (details in Section 3.1).

**Figure 5:**
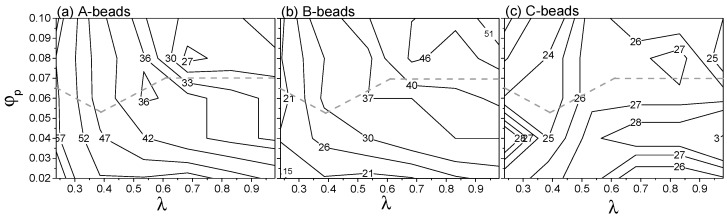
The percentage (%) of (**a**) A-; (**b**) B-; and (**c**) C-beads on the skins of aggregates (both inner- and outer-skins are included). The dashed line depicts the micelle-vesicle boundary based on the state diagram in Figure 2.

The distributions of various beads on the aggregates outer skins are presented in Supplementary Materials Figure S2b,c. How the composition of the skin (inner and outer skins combined) changes with $\varphi_{p}$ and λ is quantified in Figure 5. As λ increases, the percentage of A-beads decreases while the percentage of B-beads increases. This reflects the fact that the interactions between A- and S-beads are increasingly repulsive and the penalty of mixing increases as λ increases. The percentage of A-and B-beads is insensitive to $\varphi_{p}$ for λ 0.5. For higher values of λ, transitioning from micelles to vesicles decreases the percentage of A-beads in favour of B-beads (see Figure 5a,b). This gives A-beads better protection from S-beads and confirms that the transition of micelle to vesicle is governed by unfavourable interactions between A- and S-beads. The amount of surface A-beads are always substantial since it is the longest block of the terpolymer. The local percentage of C-beads remains relatively constant for all test conditions (see Figure 5c). This is because the C-block is the shortest block of the terpolymer. Thermodynamics and the physical constraints of the polymer architecture result in the C-block on the skins.

Examining the spatial distributions of beads on skins of aggregates show that C-beads aggregate and form domains at high λ (grey symbols, Supplementary Materials Figure S2b). Radial distribution functions of C-C beads on outer skins of spherical vesicles with high λ show peaks irrespective of $\varphi_{p}$ (see black symbols in Supplementary Materials Figure S9a,b), suggesting these C-domains form orderly patterns on the skin. Formation of ordered structures by block copolymer and terpolymer is common and is controlled by the repulsive interactions among beads [20,21,35,36]. As λ drops, the magnitude of repulsion drops and so does the orderliness of the skins morphology (see white symbols in Supplementary Materials Figure S9a,b). For A-beads and B-beads, no order is found (see Figure S9c–f).

The membrane properties were monitored by tracking the mean square displacement (MSD) of test chains for vesicles formed at $\varphi_{p}=$ 0.1, as shown in Supplementary Materials Figure S10. For all values of λ, approximately Brownian diffusion [37] can be observed over short time scales. This shows that membranes behave as viscous liquids and that the nature of the diffusion dynamics within the vesicle membranes is unaffected by the value of λ. At longer time scales, the exponent of the power law describing the MSD as function of time increases. This may be due to the test chains leaving their clusters of origin.

#### 4.5.2. Interaction with Solvent and Aggregates

In this work, interactions energies between B-B beads, B-S beads, and S-S beads are similar (see Table 1). Hence, B- and S- beads mix well. With decreasing λ, the A-S pairwise interaction also drops. Hence, it is expected that as λ decreases: (1) B-beads are more solvated, and (2) S-beads may penetrate into the A-bead inner leaflet of the bilayer membrane, i.e., the membrane may become permeable.

**Figure 6:**
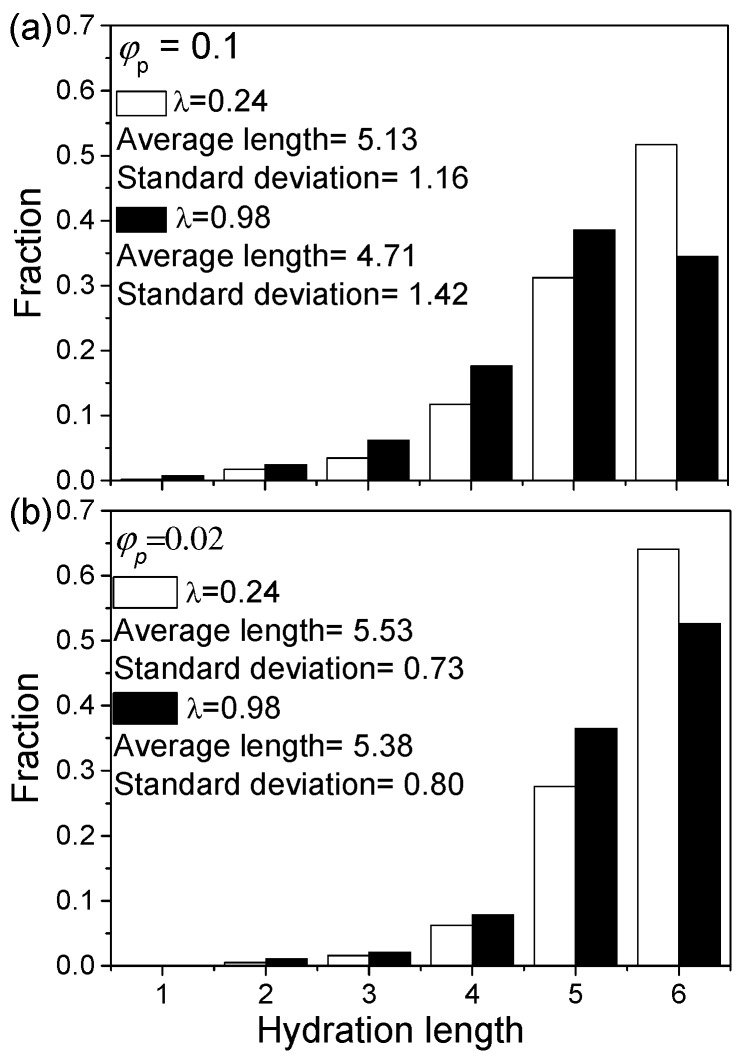
Histogram showing the length of solvated B-bead segments at various conditions from the outer skin only: (**a**) $\varphi_{p}=$ 0.1 and (**b**) $\varphi_{p}=$ 0.02.

The degree of B-bead solvation can be examined by the length of the solvated B-bead segments extending from the outer skin. The solvation of B-beads of the inner skin is excluded in this analysis because they might be affected by a confinement effect. The average solvation length and its distribution under various conditions are shown in Figure 6 (for details please see Supplementary Materials Table S1). The reduction of λ indeed sees an increase in B-bead solvation length due to a drop in energy cost for mixing. Most solvated B-bead segments are at least 4 DPD units long (see Figure 6). As $\varphi_{p}$ decreases, the average hydration length of the B-bead segments of an aggregate increases. This is due to smaller aggregates having a higher surface to volume ratio. Thus, a larger fraction of B-beads is exposed and, hence, can be solvated by S-beads.

Vesicles are frequently cited as potential cargo carriers, so their ability to encapsulate target chemicals at intended domains is important. In this work, S-beads are encapsulated at the cavity of the vesicles. The amount of S-beads encapsulated normalized by the number of terpolymer chains making up the vesicle is presented in Supplementary Materials Figure S11. An increase in λ sees a reduction in the number of encapsulated S-beads per chain in the vesicle. The amount of encapsulated S-beads per chain plateaus at high values of λ.

Assuming that the vesicles are spherical for $\varphi_{p}\;=\;0.1$, the vesicles formed at λ = 0.98 are 13% smaller than those formed at λ = 0.24. The vesicles formed at higher λ also have smaller inner cores (see Figure 4c,d and Supplementary Materials Figure S5b). Since the vesicles formed at λ = 0.98 have a core about six times smaller than those formed at λ = 0.24, they encapsulate six times fewer S-beads. Few S-beads are found in the membrane for some vesicles although they are most noticeable at λ = 0.24. This is also confirmed with the composition profiles in Figure 4d where for λ = 0.24, the profiles for S- and A-beads overlap. Small solvent molecules swelling the polymer film can act as plasticizers and improve the deformability of thin films [38]. Hence, a thicker hydration layer and more stretchable membrane leads to thinner vesicle membranes and a vesicle with a high encapsulation capability at low λ.

**Figure 7:**
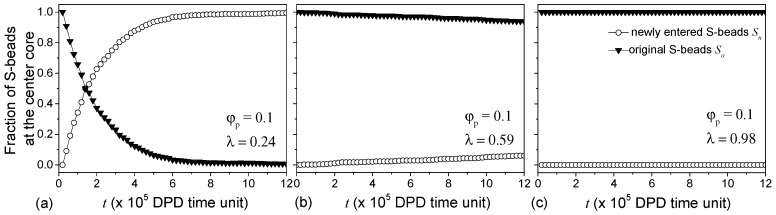
The fraction of original and new S-beads in the cores of vesicles formed at $\varphi_{p}=$ 0.1 and λ= (**a**) 0.24; (**b**) 0.59 and (**c**) 0.98. Triangles and circles correspond to the S-beads that were and were not at the cores at a reference point in time (*t* = 0) respectively.

The presence of S-beads in the inner A-bead leaflet of the membrane with low λ suggests that vesicles with low pairwise interactions improve S-bead encapsulation efficiency at the expense of the effectiveness in selectively containing S-beads to their designated compartments. The diffusion of S-beads through the vesicle membrane is examined by monitoring the exchange of S-beads in the cavity with those outside of the vesicle. Figure 7a shows for λ= 0.24, the fraction of S-beads originally in the cavity $S_{o}$ (solid triangles, Figure 7a) decreases with time. At the same time, S-beads outside of the vesicle diffuse through the membrane into the cavity, i.e., the fraction of newly-entered S-beads $S_{n}$ (open circles Figure 7a) increases. It takes roughly 250.8 ns in real-time to completely replace the original S-beads with new S-beads that were initially outside of the vesicles.

Increasing λ slows down the diffusion of S-beads across the membrane (see Figure 7b for λ = 0.59) where changes in $S_{o}$ and $S_{n}$ are small over time. Large pairwise repulsive interaction is necessary to stop such diffusion (see Figure 7c where $S_{o}\approx$ 1 and $S_{n}\approx$ 0). Similar observations were made for vesicles formed with $\varphi_{p}=$ 0.08. The results show that the permeability and the selectivity of the vesicles can be controlled by changing λ. While low λ allows for more S-beads to be encapsulated, these S-beads can diffuse out of the vesicles. Increasing λ reduces the rate of such diffusion and, hence, the choice of λ would depend on the time the vesicles are required to hold specific chemicals and can be used to control release rates.

## 5. Conclusions

In this work, the equilibrium structures of an ABC star terpolymer are examined with DPD simulations. A_12_B_6_C_2_ star terpolymers in solutions made of S beads are the model systems. The effects of concentration and strength of interactions are investigated. When the concentration of polymer $\varphi_{p}$ is very low, disorder and branched structures are found. As $\varphi_{p}$ increases, micelles and vesicles appear. The transition from micelles to vesicles occurs at the same $\varphi_{p}$ regardless of interaction energy, suggesting that a critical mass exists above which vesicles will form regardless of λ. Vesicles are generally of approximately spherical shape, whereas micelles are more irregular. The most anisotropic micelles are formed near the transition boundary. The free energy of aggregates is dominated by their surface energy with the A-S interactions being the largest contributor. For interactions among beads, A-S interactions are most important to determine equilibrium aggregate structures. The interaction energies among beads also govern the surface morphology and the size of the aggregates. It was found that an increase in repulsive interactions gives rise to aggregates that have more orderly-arranged C-domains.

In terms of encapsulation of S-beads, vesicles made of terpolymers having lower interaction energies with solvents are more efficient in terms of solvent encapsulation capability. This is because their membranes are swollen by the solvent. As a result, these membranes can be stretched more easily. The thinner membrane for these vesicles means they have more encapsulation volume per terpolymer. With more S-beads encapsulated, these vesicles are also slightly larger and have thicker hydration layers. The high efficiency for S-bead encapsulation of these vesicles comes with a price. The fact that solvent can swell the membrane of these vesicles relatively easily means encapsulated molecules can permeate through the membrane. Hence, the repulsion energy among beads should be tuned carefully to design vesicles of desired encapsulation efficiency and release rate.

## Figures and Tables

**Table 1:** Interaction parameters $a_{ij}^{0}$ (in dissipative particle dynamic (DPD) units).

Type of Beads	B	A	C	S
**B**	25.0			
**A**	38.5	25.0		
**C**	89.4	78.0	25.0	
**S**	26.0	97.9	125.0	25.0

## Supplementary Materials

The following are available online at www.mdpi.com/2073-4360/9/7/275/s1: A pdf file containing the following: **Figure S1.** How potential energy per chain changes with $\varphi_{p}$ and λ; **Figure S2.** The skin chemical compositions of vesicles formed at $\varphi_{p}=$ 0.1 (**b**) λ = 0.98; (**c**) λ = 0.24; Definitions of θ and ψ are shown in (**a**); **Figure S3.** Normalized Eigen values of moment of inertia for aggregates with $\varphi_{p}\;\geq$ 0.2 as shown in Figure 2; **Figure S4.** Contributions of A-S, B-S, and C-S interactions to total energy and skin free energy of the aggregates The hydrated layers are excluded in these calculations; **Figure S5.** (**a**) The size of aggregates $r_{a}$ and (**b**) comparing the radius of the aggregate with the radius of the cavity where S-beads are encapsulated; **Figure S6.** Radii of gyration $R_{g}$ of terpolymers and individual segments at various λ and $\varphi_{p}$; **Figure S7.** Composition profiles of aggregates excluding shells; **Figure S8.** Composition profiles of vesicles formed (including skins and hydrated layers) at $\varphi_{p}$ 0.1 and 0.08; **Figure S9.** Radial distributions of (**a**) and (**b**) C-C beads; (**c**) and (**d**) A-A beads; and (**e**) and (**f**) B-B beads on the outer skins of vesicles and micelles; **Figure S10.** (**a**) The dynamics of test chains in vesicles formed at $\varphi_{p}=$ 0.1 is presented by plotting the mean square displacement (MSD) of test chains in DPD unit against time t; **Figure S11.** The number of encapsulated S-beads normalized by the number of terpolymer chains in a vesicle; **Table S1.** Average length of solvated B-bead segments at various conditions.
