# Supplementary material for: Structure of Amphiphilic Terpolymer Raspberry Vesicles
Source: Polymers (Basel). 2017 Jul 9;9(7):275. doi: 10.3390/polym9070275 (PMC6432345; doi:10.3390/polym9070275)
Supplement: Supplementary file 1 [file polymers-09-00275-s001.pdf]

Supplementary information for 'Structure of Amphiphilic Terpolymer Raspberry Vesicles'

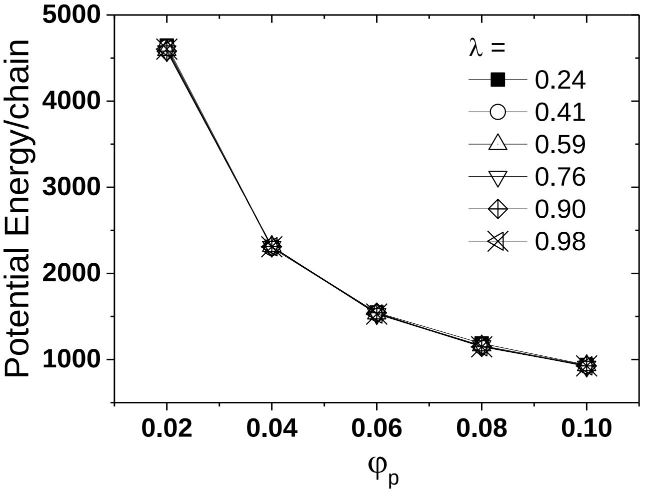

Figure S1 How potential energy per chain changes with φ_p and λ. Note micelles and vesicles are equilibrium a ggregates at low and high polymer concentration respectively. The results suggest the transition is a second ord er transition.

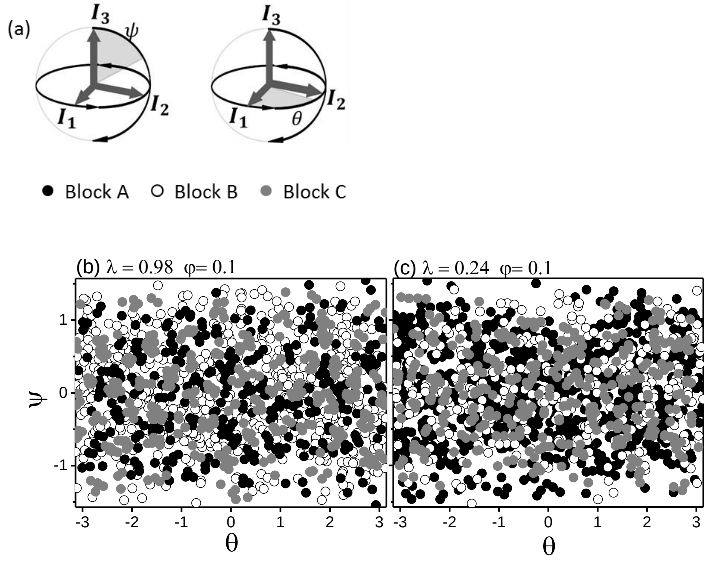

Figure S2 The chemical compositions of outer skins of vesicles formed at $\varphi_p = 0.1$ (b) $\lambda = 0.98$; (c) $\lambda = 0.24$. The horizontal and vertical axses are θ and ψ respectively. Definitions of θ and ψ are shown in (a).

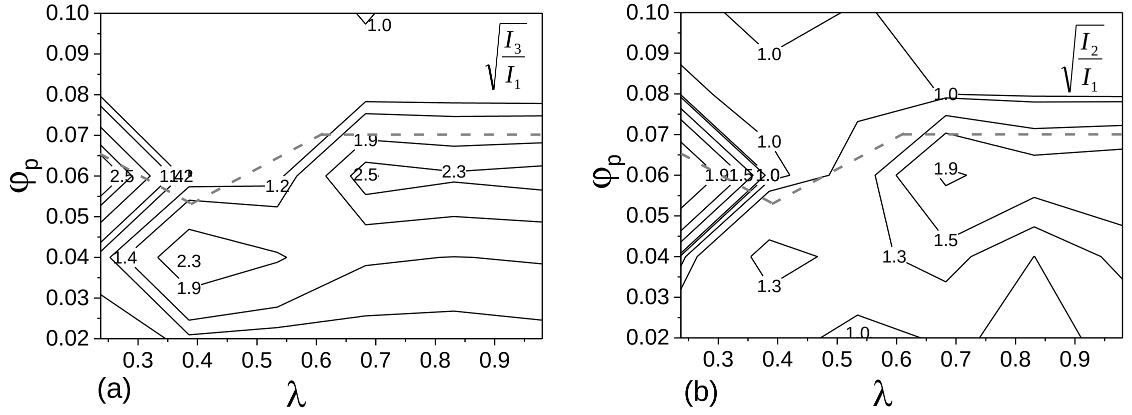

Figure S3 Normalised Eigen values of moment of inertia for aggregates with $\varphi_p \geq 0.2$ as shown in Figure 2. The hydrated layers are excluded in these calculations. A spherical aggregate has $\sqrt{\frac{I_2}{I_1}} = \sqrt{\frac{I_3}{I_1}} = 1$. Grey dash line depicts the boundary above which the aggregates transform from micelles to vesicles.

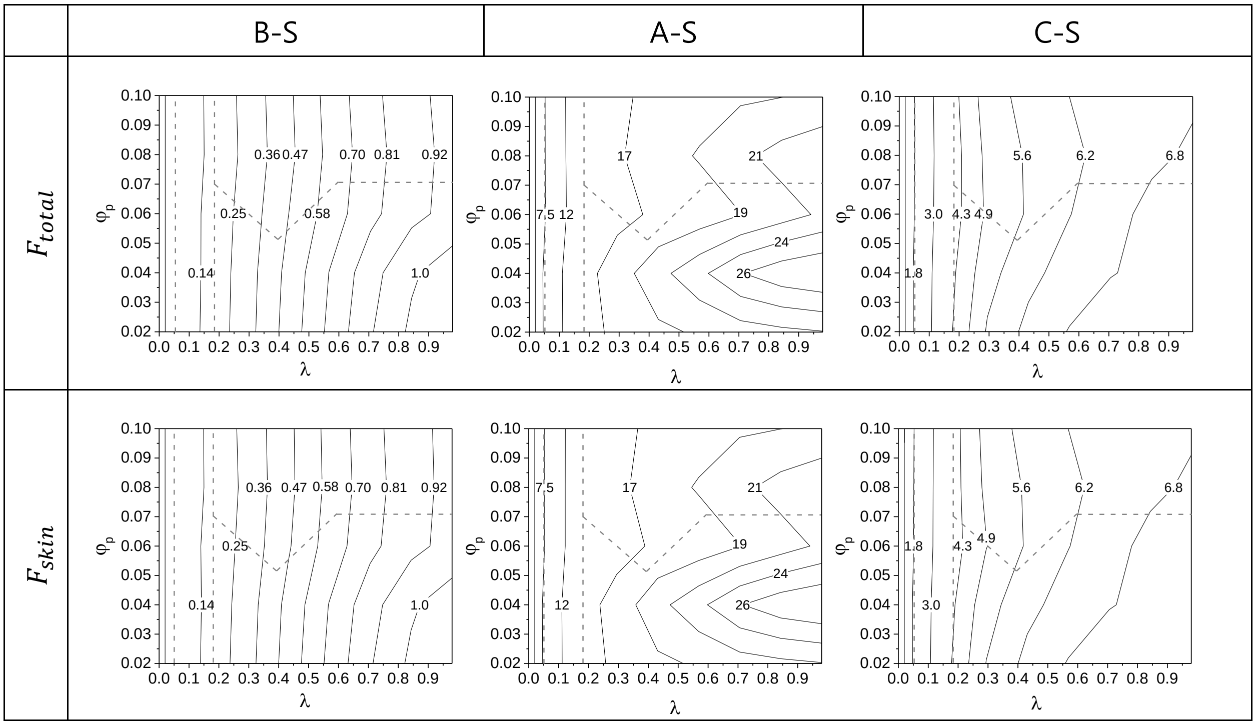

Figure S4 Contributions of A-S, B-S, and C-S interactions to total energy F_{total} and skin free energy F_{skin} of the aggregates.

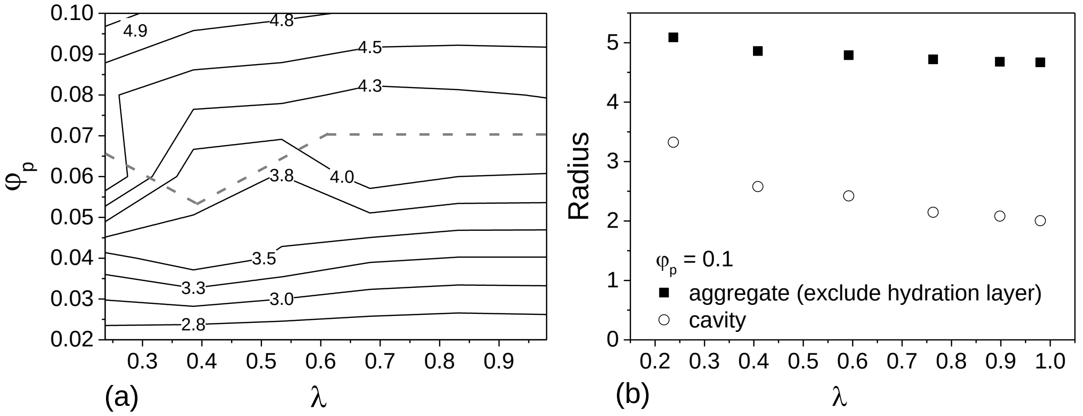

Figure S5 (a) The size of aggregates r_a. The hydrated layers are excluded in these calculations. Grey dash li ne depicts the boundary above which the aggregates transform from micelles to vesicles. (b) Comparing the rad ius of the aggregate with the radius of the cavity where S-beads are encapsulated.

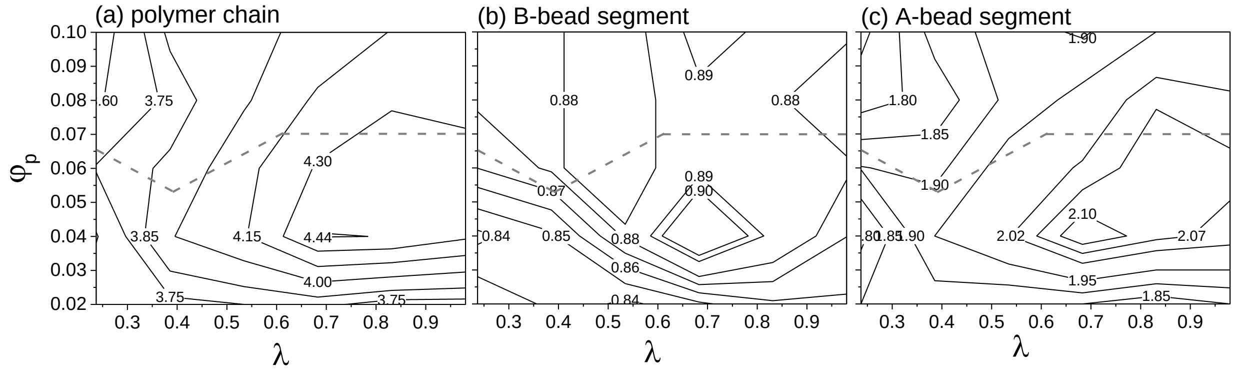

Figure S6 Radii of gyration R_g of terpolymers and individual segments at various λ and φ_p. Dash line depi cts the micelle-vesicle boundary based on the state diagram in Figure 2

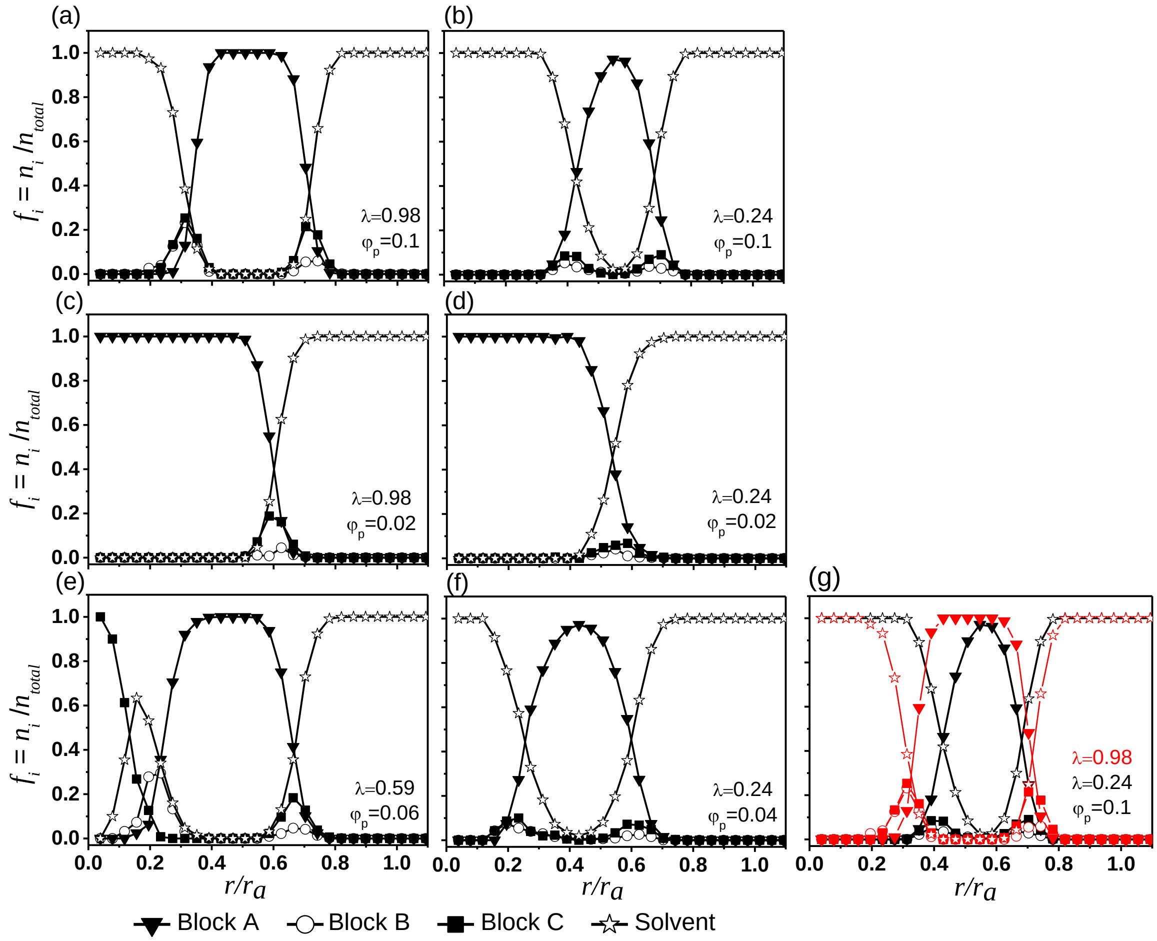

Figure S7 (a) to (f) Composition profiles of aggregates (excluding shells); (g) a comparison of composition profiles between Figure S7(a) and S7(b).

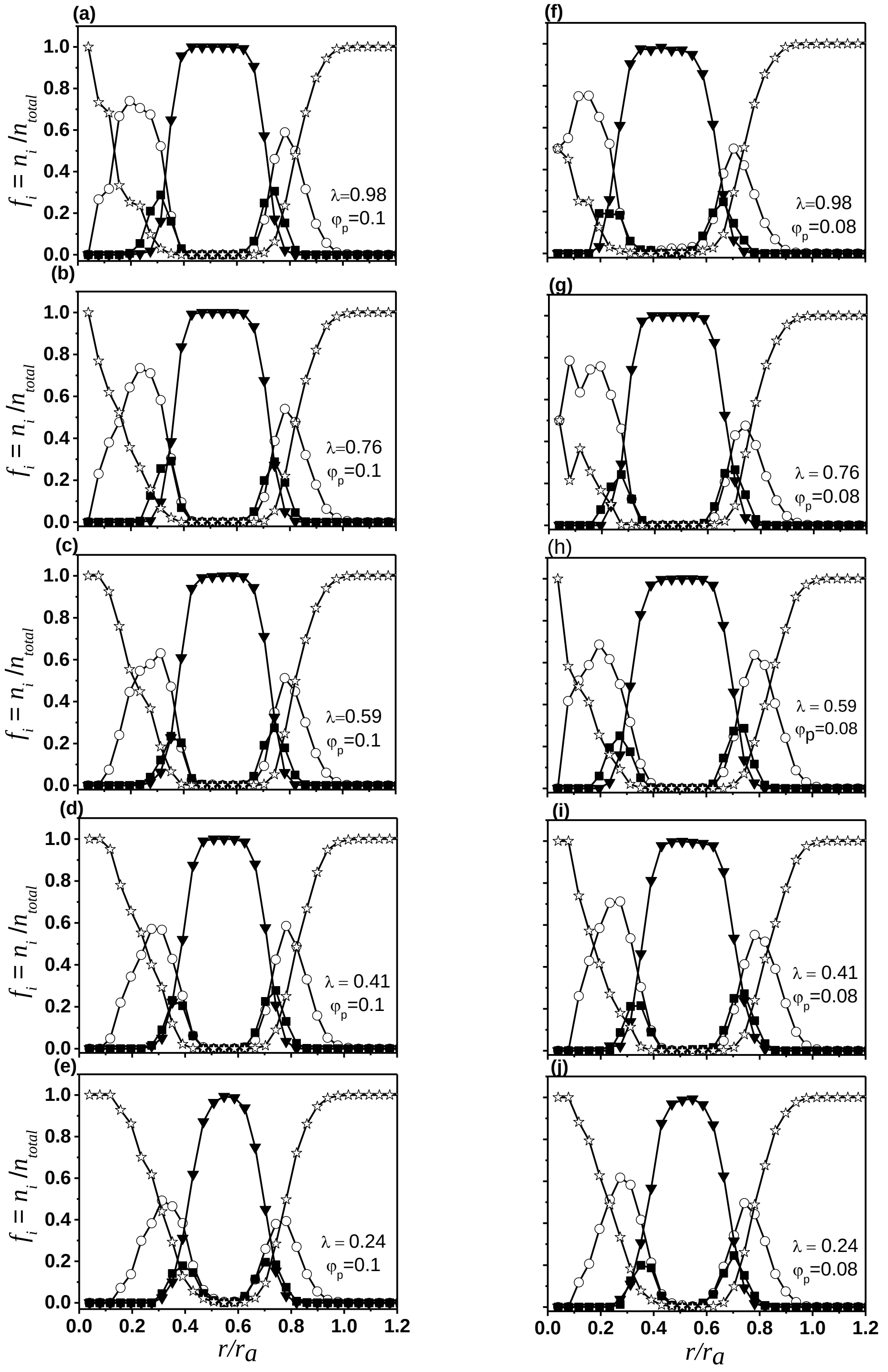

Figure S8 Composition profiles of vesicles formed (including skins and hydrated layers) at $\varphi_p = 0.1$ and 0.08.

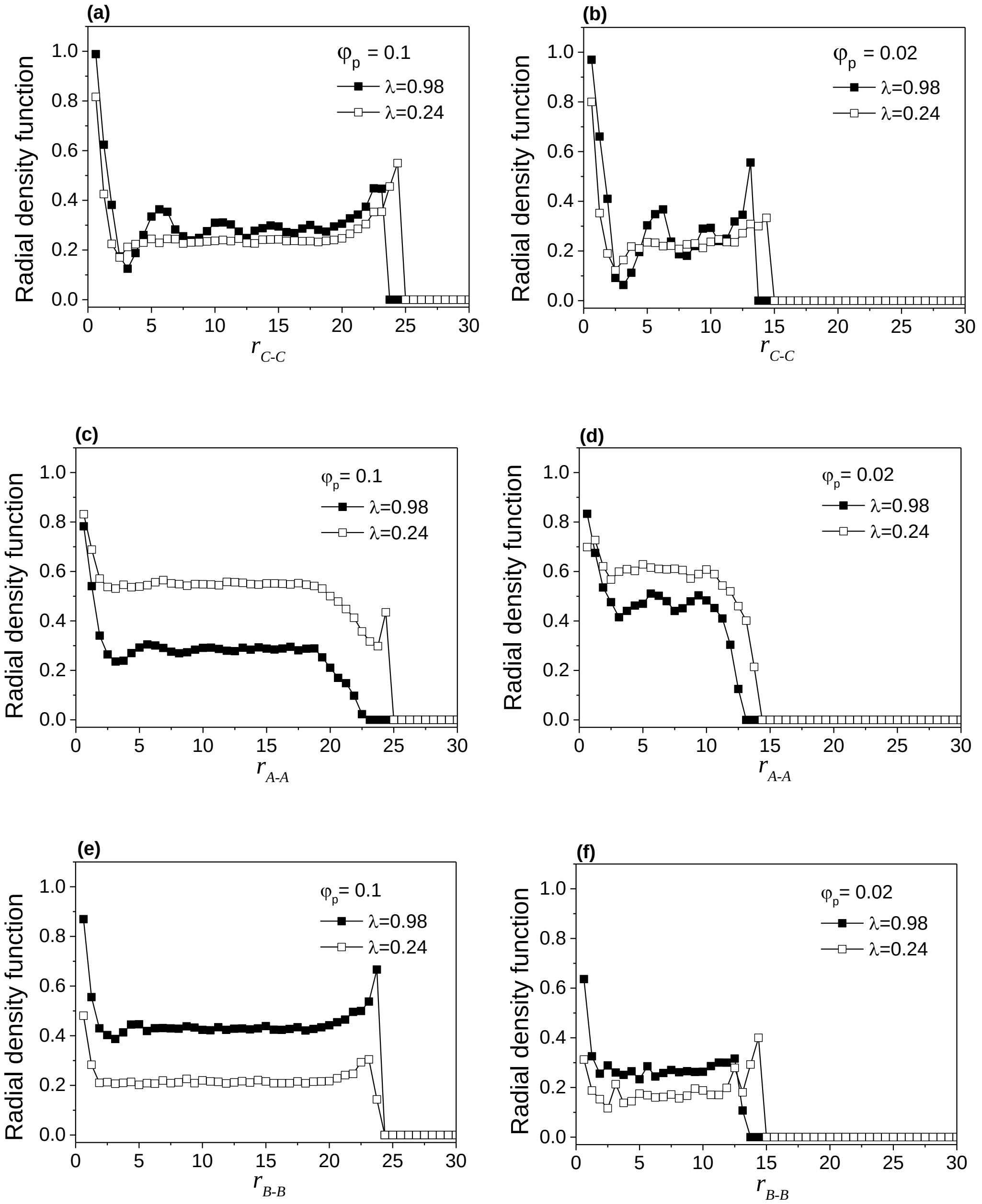

Figure S9 Radial distributions of (a) and (b) C-C beads; (c) and (d) A-A beads; and (e) and (f) B-B beads on the outer skins of vesicles and micelles. The horizontal axis is the distance between beads in DPD units. The vertical axis is the fraction of bead i, i.e. for each bin, f_{i-i} = Number of i neigbour/Number of total neigbour around a i-bead.

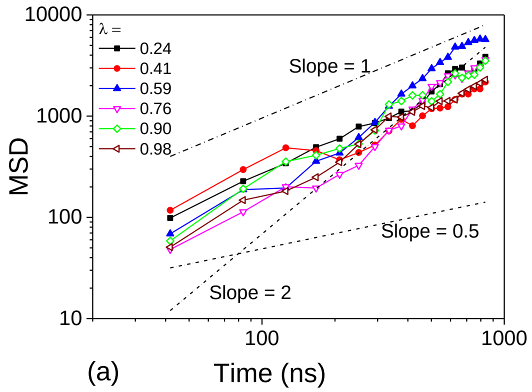

Figure S10 (a) The dynamics of test chains in vesicles formed at φ_p = 0.1 is presented by plotting the mean square displacement (MSD) in DPD unit against time *t*. The dash lines are for guidance only. At time t = 0, equilibrated vesicles have already been formed.

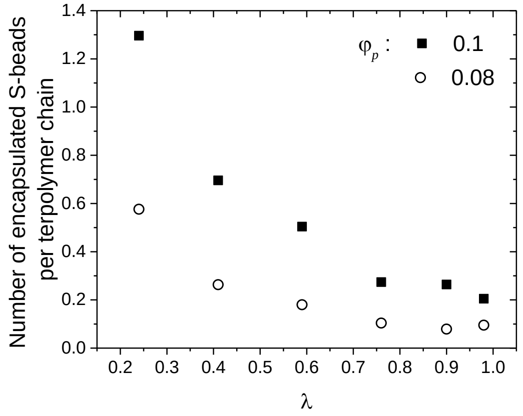

Figure S11 The number of encapsulated S-beads per terpolymer chain in vesicles formed at various conditions.

		Average hydration length of hydrated O-bead segments					
φ_P	**0.1**	5.13 (1.16)	4.99 (1.25)	4.89 (1.26)	4.85 (1.37)	4.80 (1.30)	4.71 (1.42)
	**0.08**	5.18 (1.19)	5.11 (1.16)	4.98 (1.25)	4.86 (1.36)	4.86 (1.28)	4.84 (1.36)
	**0.06**	5.31 (1.06)	5.25 (1.21)	5.08 (1.32)	5.02 (1.04)	5.05 (1.06)	4.96 (1.10)
	**0.04**	5.38 (1.13)	5.34 (0.90)	5.19 (1.03)	5.22 (0.86)	5.18 (0.92)	5.14 (0.97)
	**0.02**	5.53 (0.73)	5.43 (0.79)	5.51 (0.75)	5.43 (0.74)	5.32 (0.76)	5.37 (0.80)
		**0.24**	**0.41**	**0.59**	**0.76**	**0.90**	**0.98**
		λ					

Table S1. Average length of solvated B-bead segments at various conditions. Values in brackets are standard deviations.
